# Molecular dissection of *Secale africanum* chromosome 6R^afr^ in wheat enabled localization of genes for resistance to powdery mildew and stripe rust

**DOI:** 10.1186/s12870-020-02351-1

**Published:** 2020-03-31

**Authors:** Guangrong Li, Lingrong Tang, Yan Yin, Ahui Zhang, Zhihui Yu, Ennian Yang, Zongxiang Tang, Shulan Fu, Zujun Yang

**Affiliations:** 1 Center for Informational Biology, School of Life Science and Technology, University of Electronic and Technology of China, Chengdu, 611731 Sichuan China; 2 Crop Research Institute, Sichuan Academy of Agricultural Sciences, Chengdu, 610066 Sichuan China; 3 Province Key Laboratory of Plant Breeding and Genetics, Sichuan Agricultural University, Chengdu, 611130 China

**Keywords:** Adult plant resistance, Powdery mildew, *Secale africanum*, Stripe rust, Wheat

## Abstract

**Background:**

Introgression of chromatin from *Secale* species into common wheat has for decades been a successful strategy for controlling the wheat diseases. The wild *Secale* species, *Secale africanum* Stapf., is a valuable source for resistance to foliar disease of wheat. A wheat-*S. africanum* chromosome 6R^afr^ substitution line displayed resistance to both powdery mildew and stripe rust at the adult-plant stage.

**Results:**

Wheat-*S. africanum* chromosome 6R^afr^ deletion and translocation lines were produced and identified by sequential non-denaturing fluorescence in situ hybridization (ND-FISH) using multiple Oligo-based probes. Different ND-FISH patterns were observed between *S. cereale* 6R and *S. africanum* 6R^afr^. With reference to the physical map of the draft genome sequence of rye inbred line Lo7, a comprehensive PCR marker analysis indicated that insertions and deletions had occurred by random exchange between chromosomes 6R and 6R^afr^. A survey of the wheat- *S. africanum* 6R^afr^ lines for disease resistance indicated that a powdery mildew resistance gene(s) was present on the long arm of 6R^afr^ at FL0.85–1.00, and that a stripe rust resistance gene(s) was located in the terminal region of 6R^afr^S at FL0.95–1.00. The wheat-*S. africanum* 6R^afr^ introgression lines also displayed superior agronomic traits, indicating that the chromosome 6R^afr^ may have little linkage drag in the wheat background.

**Conclusions:**

The combination of molecular and cytogenetic methods allowed to precisely identify the chromosome rearrangements in wheat- *S. africanum* 6R^afr^ substitution, deletion and translocation lines, and compare the structural difference between chromosomes 6R and 6R^afr^. The wheat- *S. africanum* 6R^afr^ lines containing gene(s) for powdery mildew and stripe rust resistance could be used as novel germplasm for wheat breeding by chromosome engineering.

## Background

Powdery mildew of wheat, caused by *Blumeria graminis* f. sp. *tritici* (Bgt), and stripe rust (yellow rust), caused by *Puccinia striiformis* f. sp. *tritici* (Pst), are important diseases that occur in most wheat-growing regions. The areas affected by powdery mildew and stripe rust epidemics have increased under both rain-fed and irrigated high-input conditions in China, and also in many other countries in recent years [1, 2]. Cultivation of resistant cultivars is economical and environmentally friendly compared to chemical control. Considerable numbers of powdery mildew and stripe rust resistance genes have been identified and used in wheat breeding [3–5]. However, the effectiveness and commercial usefulness of these genes is often reduced due to emergence of new pathogen races. Race non-specific resistance and adult plant resistance (APR) genes, such as the multipathogen APR genes *Lr34/Yr18/Sr57/Pm38* [6] and *Lr67/Yr46/Sr55/Pm46* [7] are considered more durable than race specific resistance. The APR genes originated from the related species to wheat were also taken into consideration [8].

Cultivated rye (*Secale cereale* L.) has long been a valuable source of potentially useful genes for wheat improvement, which provided rich diversity for disease resistance gene introgression [8, 9]. Two of the most successful translocations were 1BL.1RS and 1AL.1RS, which carry powdery mildew genes *Pm8* and *Pm17*, respectively. Further molecular clarification of *Pm8* and *Pm17* revealed that they were orthologous resistance genes with different evolutionary history [10, 11]. Friebe et al. [12] localized the powdery mildew resistance gene *Pm20*, which was derived from the chromosome 6RL of *S. cereale* cv. Prolific, which is currently still effective in China [13]. Chromosome 6RL derived from *S. cereale* cultivars German White [13], JZHM [14], and Kustro [15, 16] also possess gene(s) for powdery mildew resistance. Another new powdery mildew resistance gene *Pm56*, which originated from Chinese native rye cultivar (cv.) QL [17], was recently mapped to the subtelomeric region of chromosome 6RS. However, the reports on the occurrence of stripe rust resistance genes derived from *Secale* species are limited. Chromosome 1RS in many wheat cultivars grown worldwide carry stripe rust resistance gene *Yr9* [4]; however, that gene is no longer effective in most locations. Recently, a new stripe rust resistance gene *Yr83* from 6R was reported [18], and chromosomes 1R and 4R with effective stripe rust resistance have been also incorporated into wheat in China [19, 20]. The potentially novel *Yr* genes on these chromosomes from different rye donors need further evaluation.

The wild species of *Secale* and related genera are considered potentially new sources of variation for wheat breeding purposes [21]. In order to introduce novel disease resistance genes from the wild *Secale* species *S. africanum* into common wheat, we initiated a cross between a *Triticum turgidum–S. africanum* amphiploid and cultivated wheat [22, 23] and developed a large number of wheat-*S. africanum* introgression lines [24]. Wheat-*S. africanum* substitution lines involving chromosomes 1R^afr^ [25], 2R^afr^ [26, 27], 5R^afr^ [28] and 6R^afr^ [29] with desirable agronomic traits were developed. These lines permitted the localization of genes on specific *S. africanum* chromosomes.

In the present study, we identified wheat - *S. africanum* chromosome 6R^afr^ substitution, deletion and translocation lines and physically localized gene(s) for powdery mildew and stripe rust resistance. The results also provided the opportunity to identify chromosomal rearrangements in the *Secale* genus by comparative molecular and cytogenetic analyses*.*

## Results

### Identification of *S. africanum* 6R^afr^ and *S. cereale* 6R using ND-FISH

**Fig. 1:**
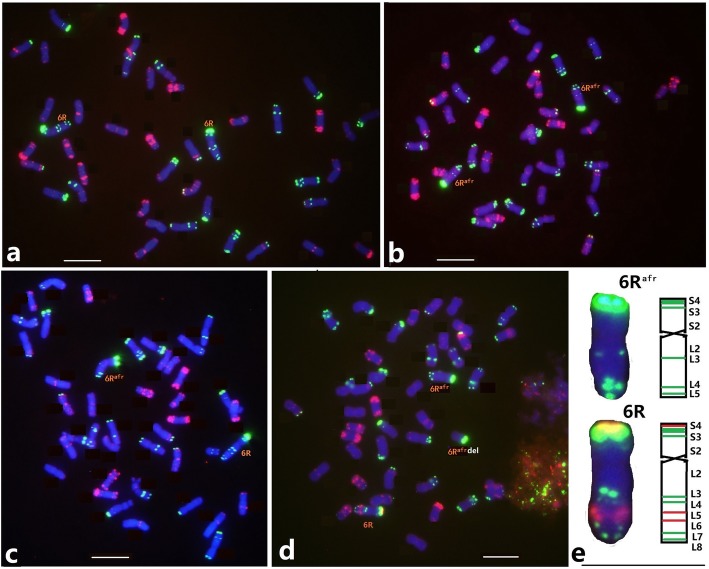
ND-FISH of wheat-rye 6R **a** and wheat-*S. africanum* 6R^afr^**b** substitution lines and their hybrid progenies **c**, **d**. The probes Oligo-pSc119.2 (green) + Oligo-pTa535 (red) **a** to **c**, and Oligo-pSc119.2 (green) + Oligo-pTa535 (red) + Oligo-pSc200 (red) **d** were used for FISH. Seven regions of the 6R^afr^ chromosome and 10 for 6R chromosome are indicated in the ideogram **e**. Green and red bands on chromosome 6R and 6R^afr^**e** represent Oligo-pSc119.2 and Oligo-pSc200 signals, respectively. Bar, 10 μm

Non-denaturing FISH using probes Oligo-pSc119.2 and Oligo-pTa535 was conducted to characterize mitotic metaphase spreads of the wheat-*S. cereale* 6R (6D) line DS6R [18] and wheat-*S. africanum* 6R^afr^ (6D) line HH41 [29]. FISH of line DS6R showed that telomeric region of chromosome 6RS had a single strong Oligo-pSc119.2 signal, whereas the 6RL had four prominent signal sites, two interstitial and two sub-telomeric (Fig. 1a). The *S. africanum* 6R^afr^ in HH41 had a strong Oligo-pSc119.2 signal at the telomeric region of the short arm, two signals in the terminal and sub-terminal regions of the long arm, and a faint hybridization site close to the centromere (Fig. 1b). FISH of hybrids between HH41 and DS6R revealed a length difference between 6R^afr^ and 6R (Fig. 1c); 6RL was about 15% longer than 6R^afr^L based on observations of 20 cells. Furthermore, a strong hybridization Oligo-pSc200 signal was present on 6RS, and two Oligo-pSc200 sites were located between the interstitial and sub-telomeric regions of 6RL, whereas chromosome 6R^afr^ was devoid of Oligo-pSc200 signals (Fig. 1d). Chromosomes 6R^afr^ and *S. cereale* 6R can thus be easily distinguished in the wheat background. The comparative FISH maps of the probes Oligo-pSc119.2 and Oligo-pSc200 on chromosomes 6R and 6R^afr^ are shown in Fig. 1e. It is probable that the presence or absence of pSc200-like sequences may cause the length differences between 6R and 6R^afr^.

### Identification of a 6R^afr^ deletion and translocations using FISH

**Fig. 2:**
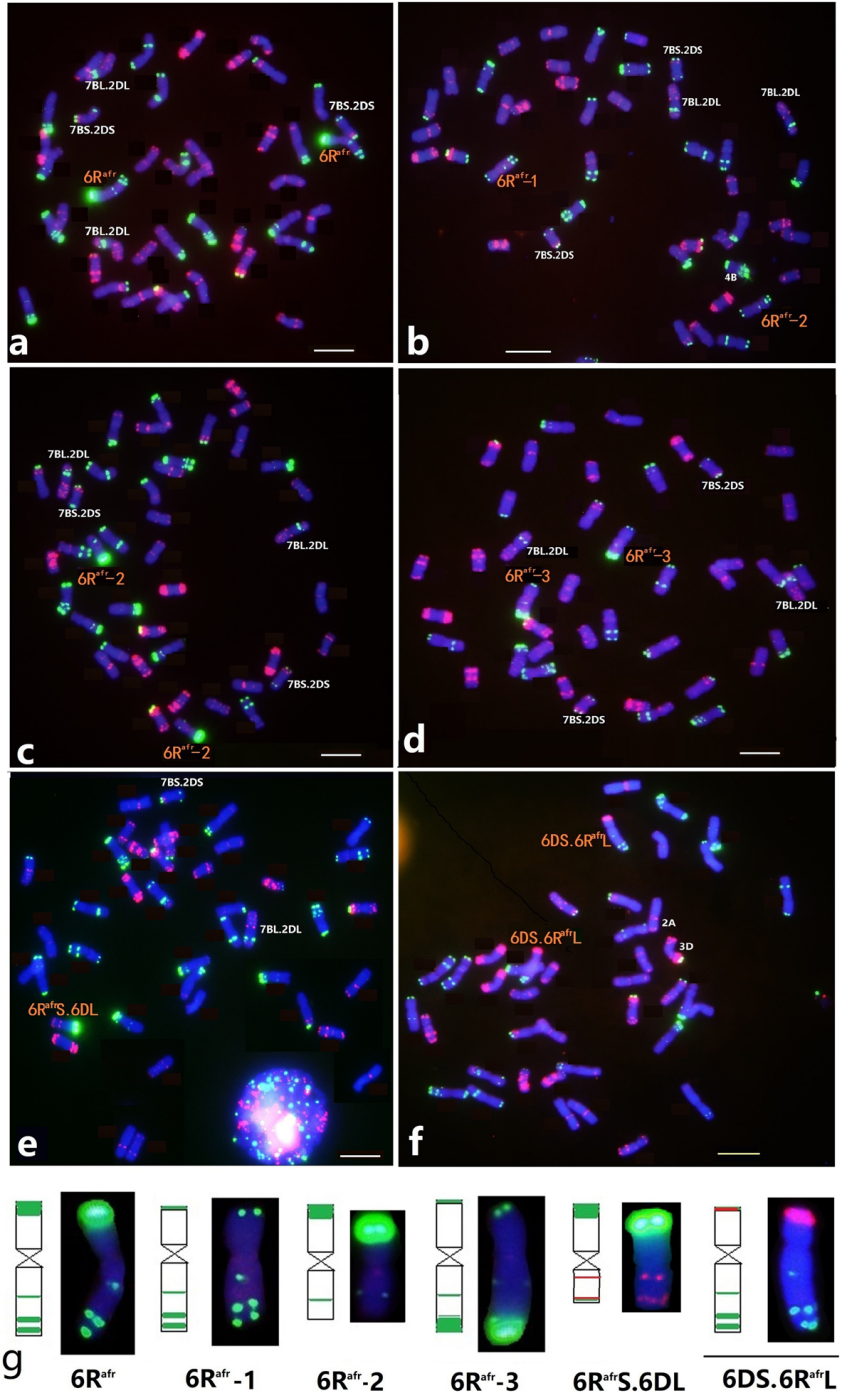
FISH of wheat-*S. africanum* chromosome 6R^afr^ (6D) substitution lines M26-B **a**, r522-D **b**, r1870-C **c**, r600-E **d**, H136 **e**, and H86 **f**. Oligo-pSc119.2 repeats are green and Oligo-pTa535 repeats are red. Bar, 10 μm

**Fig. 3:**
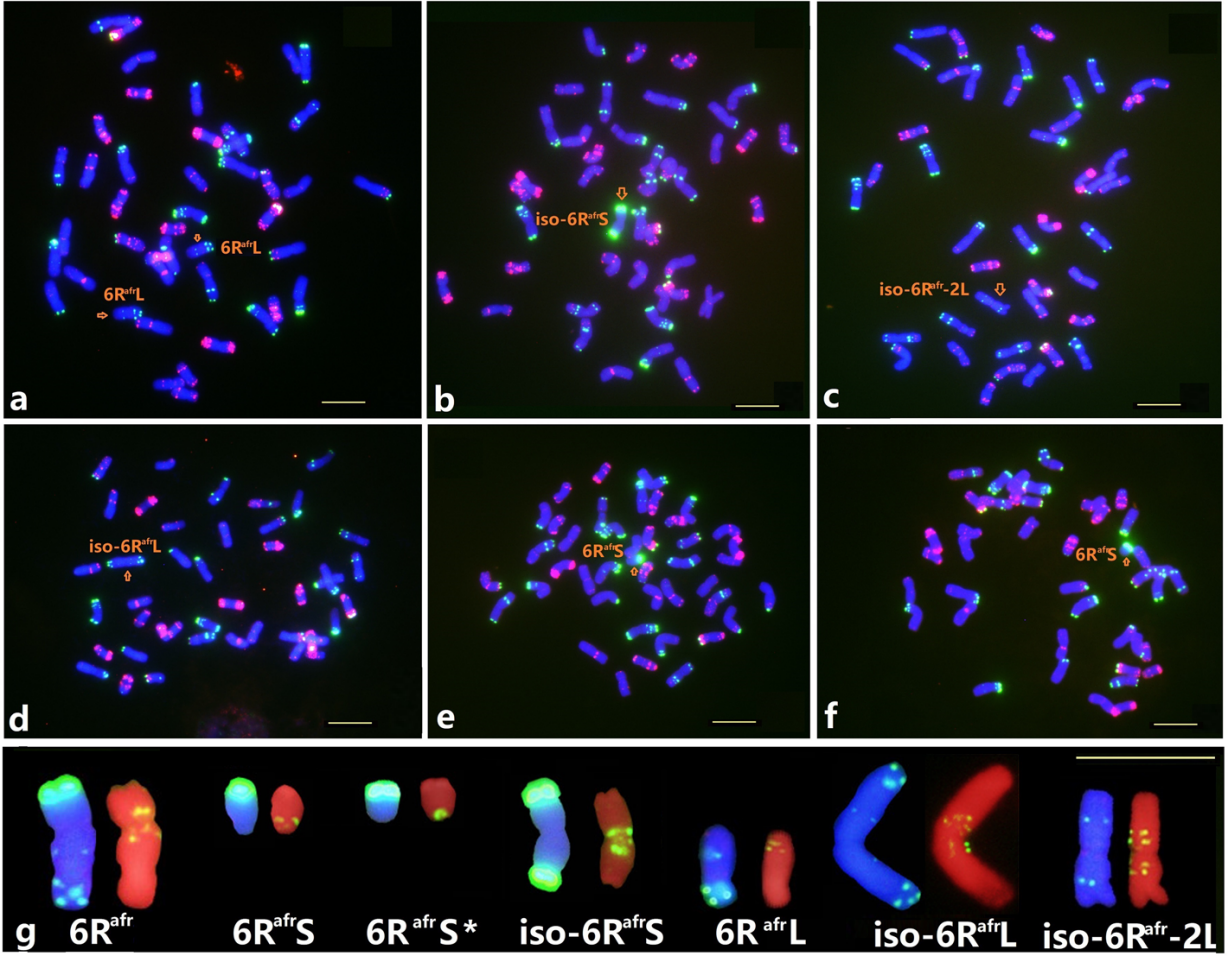
FISH of wheat-*S. africanum* 6R^afr^ lines among F_4_ progenies of HH41 × MY11 hybrids. Oligo-pSc119.2 (green) and Oligo-pTa535 (red) probes were used for FISH **a** to **f**. Line H536 **a** contains a pair of 6R^afr^L telosomes, H297 **b** has a monosomic iso-6R^afr^S chromosome, H183 **c** contains a monosomic iso-6R^afr^-2 L chromosome in which 6R^afr^L lost the two terminal Oligo-pSc119.2 sites, H384 **d** contains a iso-6R^afr^L monosome, H316 **e** contains a 6R^afr^S telosome, and H320 **f** contains a deleted 6R^afr^S telosome. FISH karyotypes **g** of chromosome 6R^afr^ and deletion chromosomes are shown after hybridization with probes Oligo-pSc119.2 (left) and Oligo-(CAA)_7_ (right), respectively. Arrows indicate the *S. africanum* chromosomes. Bar, 10 μm

A deletion line, translocation lines involving chromosome 6R^afr^ and wheat chromosomes identified among progenies of irradiated line HH41, and in progeny from the hybrid between HH41 and wheat line MY11 were screened by ND-FISH using Oligo-pSc119.2 and Oligo-pTa535. Four structural variants of 6R^afr^ were identified among 75 M_4_ lines derived from the irradiated HH41 line and named M26-B, r522-D, r1870-C and r600-E (Fig. 2). All four lines contained two copies of a 7B-2D reciprocal translocation. Line M26-B contained a pair of normal 6R^afr^ chromosomes substituting for chromosome 6D, but a reciprocal 7BS.2DS and 2DL.7BL was present in the wheat background (Fig. 2a). The FISH physical map of 6R^afr^ following hybridization with Oligo-pSc119.2 (Fig. 1e) showed that line r522-D had a deletion of the distal end of 6R^afr^S (named 6R^afr^-1, Fig. 2b) and line r1870-C contained a deletion of the terminal end of 6R^afr^L (named 6R^afr^-2, Fig. 2c). Line r600-E had a pair of modified 6R^afr^ chromosomes on which the strong terminal Oligo-pSc119.2 region was re-located to the end of the long arm, suggesting that a large inversion had occurred (named 6R^afr^-3, Fig. 2d). In addition, a monosomic 6R^afr^S.6DL translocation (line H136, Fig. 2e), and a disomic 6DS.6R^afr^L translocation (line H86, Fig. 2f) were recovered among 286 plants derived from F_4_ families from the HH41 × MY11 cross. Lines disomic for 6R^afr^L (H536), monosomic for 6R^afr^S (H316), deleted for 6R^afr^S (H320), isochromosomal iso6R^afr^S (H297), iso6R^afr^L (H384), and iso6R^afr^-2 L (H183) were also identified from these families (Fig. 3a-f). The FISH karyotypes of the broken and rearranged 6R^afr^ chromosome were further detected with a Oligo-(CAA)_7_ probe (Fig. 3g). All types of the 6R^afr^ deletion and translocation lines (Fig. 2 and Fig. 3) were confirmed by sequential FISH using the *Secale*-specific probe Oligo-1162 [30].

### Physical localization of 6R^afr^S specific markers

Li et al. [15] localized 20 specific length amplified fragment (SLAF) markers to *S. cereale* cv. Kustral 6R^Ku^S in a wheat background. These markers were tested on the 6R^afr^ deletion lines, but only 12 showed identical lengths of 6R-specific amplification products from M26-B, r522-D, r1870-C and r600-E. The 6R^afr^S.6DL line H136 showed amplification of the same 12 primer pairs, indicating that those markers were physically located in the S2-S3 region of 6R^afr^S (Fig. 1). Markers KU555 and KU226 on 6R^Ku^S were amplified in line H86 with 6DS.6R^afr^L and are therefore located on 6R^afr^L. These results indicated that the centromeric regions were structurally rearranged between 6R^Ku^ and 6R^afr^.

**Fig. 4:**
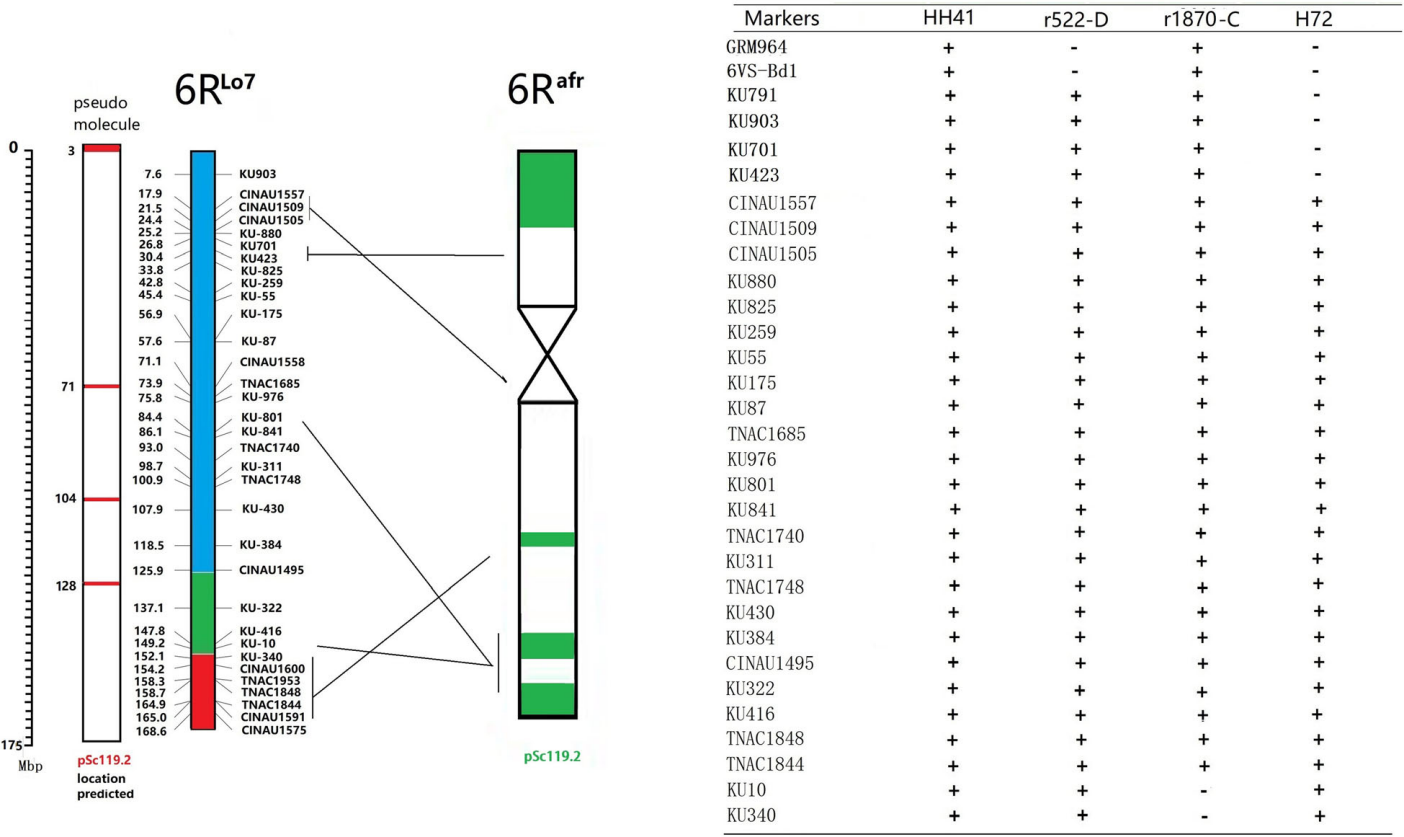
Physical location of *S. africanum* 6R^afr^ chromosome-specific markers Red lines inside the box indicate predicted pSc119.2 repeat regions (left); colors inside the box (middle) indicate likely homologous 6R regions of linkage groups 6 (blue), 3 (green) and 7 (red). The green lines on the chromosome at the right mark predicted Oligo-pSc119.2 hybridization sites. The “+” represent amplification, while “-” represent no amplification of 6R^afr^ specific bands

Markers GRM964 [31] and 6VS-Bd1 [32] were amplified in M26-B, r1870-C, r600-E and H136, but there was no amplification in r522-D and 6R^afr^L lines. Therefore these two markers are probably located in the telomeric S3 to S4 region of 6R^afr^ (Fig. 4 and Supplementary Table 1). In the assembled pesudo-molecules of *S. cereale* cv. Lo7, marker GRM964 was genetically mapped to the telomeric region of 2RS [31]. Therefore, the marker and FISH data suggested that the telomeric Oligo-pSc119.2-rich region of S3 to S4 in 6R^afr^S (Fig. 1) was syntenic to the distal region of 2R^Lo7^S.

### Localization of molecular markers on chromosome 6R^afr^L

Qiu et al. [33] localized 190 markers specific for 6R^Ku^L in Kustro rye. These markers were used to amplify DNA from the present 6R^afr^ derivatives. Only 66 of the 6R^Ku^L-specific markers generated identical amplicons in 6R^afr^(6D) substitution line HH41; the remaining 124 markers failed to amplify. Qiu et al. [33] physically located the 66 markers to three regions of 6R^Ku^L; 35 (59%) of 60 markers in region I, 16 (35.6%) of 45 in region II, and 15 of 85 (17.6%) in region III showed specific amplification for 6R^afr^L. This suggested that regions close to the centromere in both 6R^Ku^ and 6R^afr^ are closely homologous, whereas the interstitial to sub-telomeric regions were more divergent. The greatest divergence occurred in the telomeric regions.

The DNA sequences of the 66 markers for 6R^Ku^L were compared using the BLAST algorithm to the pseudo-molecules of 6R^Lo7^, and 16 of these markers were physically located in the region covered by the pseudo-molecules of the Lo7 genome (Supplementary Table 1). To add more markers to the physical map of 6R^afr^, 190 PLUG markers [34] and 321 CINAU markers [35] were assayed in amplified DNA from the 6R^afr^ deletion and translocation lines. The M26-B, r1870-C and H72 lines were also used for the amplification of the markers to determine the physical locations of these markers in chromosome 6R^afr^L. Thirty nine markers were physically mapped to L2 to L4 regions of 6R^afr^L (Supplementary Table 1 and Fig. 4). Three markers, KU-810, KU-10 and KU-340 mapped to the L4 to L5 regions of 6R^afr^L (Fig. 1).

Twenty four PLUG and CINAU markers corresponding to syntenic regions of wheat homoeologous chromosome groups 6, 3, 7 were physically located in regions corresponding to the pseudo-molecule in the draft Lo7 sequences (Supplementary Table 1). The results were consistent with the physical locations in the assembled genes in 6R^Lo7^L [36] and homologous regions of the wheat genome [37]. The distribution of pSc119.2 repeats was also predicted on the pseudo-molecule of 6R^Lo7^ based on data from the B2DSC web server at http://mcgb.uestc.edu.cn/b2dsc [38]. The pSc119.2 repeats were physically located on three regions of 71-72 Mb, 104-105 Mb and 128-129 Mb on chromosome 6R^Lo7^ (Fig. 4). A comparison between the physical locations of the molecular and cytogenetic markers clearly indicated structural changes among 6R^Lo7^L, 6R^Ku^L and 6R^afr^L.

#### RNAseq of 6R^afr^ lines

**Fig. 5:**
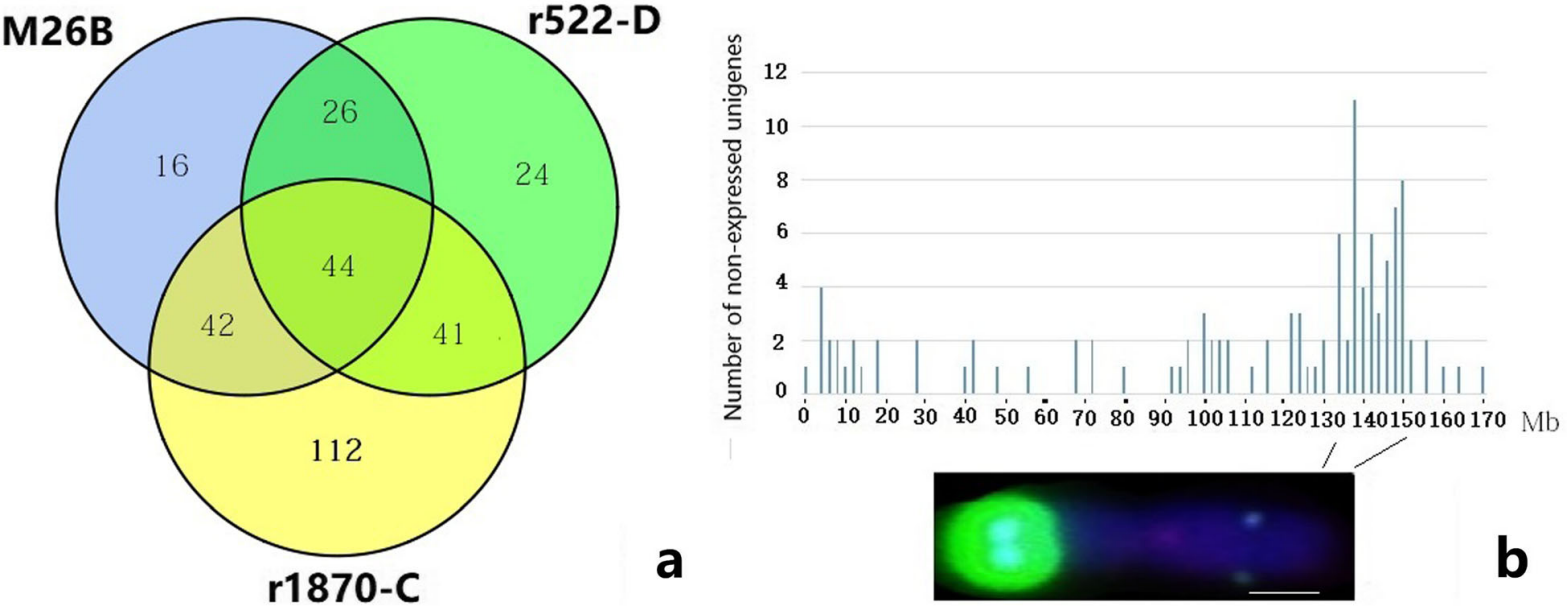
Number of non-expressed unigenes among the 6R^afr^ lines **a** and physical location in r1870-C compared with 6R^Lo7^**b**. Bar, 2 μm

In order to further characterize the deleted segment of 6R^afr^L in line r1870-C, a transcriptome analysis was performed to compare lines M26-B, r522-D, r1870-C and wheat parent MY11. A total of 42,572,656, 41,584,320, 44,426,580, and 44,932,204 reads were obtained for M26-B, r522-D, r1870-C and MY11, respectively. A sequence comparison revealed that about 80.59–81.66% reads were mapped on CS genome. Many fewer reads (34.9–36.8%) mapped to chromosome 6D in the 6R^afr^ lines than to 6D (81.9%) in MY11 indicating a lack of 6D in lines M26-B, r522-D, and r1870-C. The unigenes expressed in r26B, r522-D and r1870-C from the transcriptome data were also submitted to a search for genes annotated in pseudo-molecules of chromosome 6R^Lo7^ [36]. A total 2919 annotated transcripts covering 172 Mb specific for 6R^Lo7^ were identified. The missing transcripts (with FPKM value = 0) were compared among the lines M26-B, r522-D and r1870-C (Fig. 5a) and 112 transcripts were missing in r1870-C. Based on the distribution of the non-expressed transcripts in the physical map of 6R^Lo7^ (Fig. 5b), the corresponding regions of ~ 132–150 Mb of 6R^Lo7^ were missing transcripts in r1870-C. Since the missing transcripts could be due to silencing or deletion of these regions, the results suggested that the deletion of r1870-C chromosome 6R^afr^ might be homologous to the genomic region of 132–150 Mb in 6R^Lo7^ (Fig. 5).

### Powdery mildew and stripe rust reactions

**Fig. 6:**
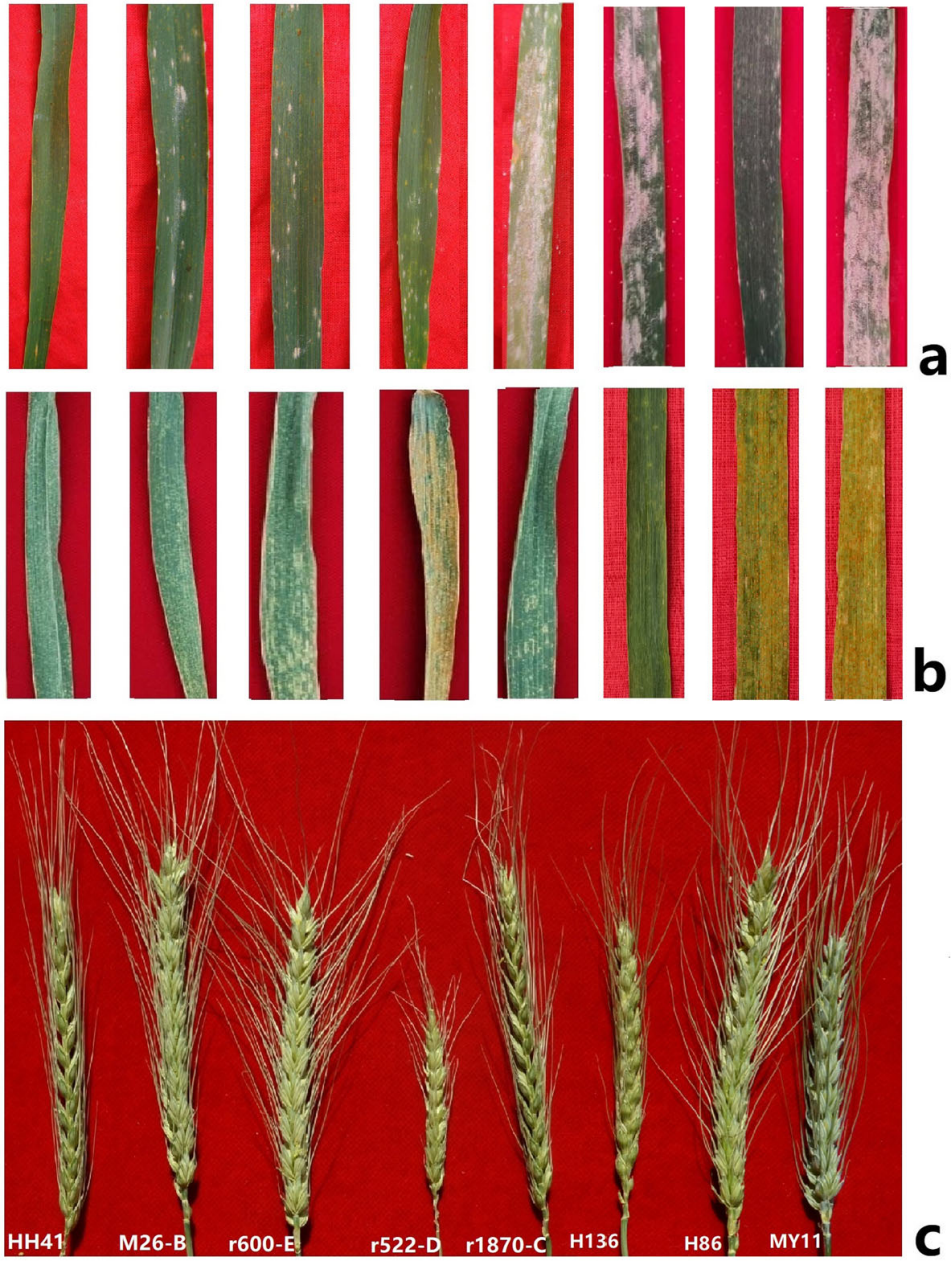
Powdery mildew **a** and stripe rust **b** reactions, and spikes morphology **c** of wheat – 6R^afr^ lines HH41, M26-B, r600-E, r522-D, r1870-C, H86, H136, and MY11

All 6R^afr^ lines were susceptible to *Bgt* isolates at the three-leaf seedling stage with infection types 3–4. The wheat parent MY11 was highly susceptible at all stages of growth. However, HH41 (IT = 0;, DS = 5) was fully resistant from the five-leaf stage and was considered to have adult-plant resistance (APR). Lines r1870-C and H136 were highly susceptible (IT = 4, DS = 85–90). The low infect type (IT = 0–1) and mean disease severity (DS = 18.6) of lines M26-B, r522-D, r600E, and H86 were characterised by necrotic areas without sporulation (Table 1, Fig. 6). This result indicated that the L4 to L5 segment (~FL 0.85–1.00) of *S. africanum* 6R^afr^L carried a gene(s) for powdery mildew resistance.

**Table 1:** Agronomical traits and disease resistance of wheat cv. MY11 and wheat-*S. africanum* 6R^afr^ derivatives

Genotype	Plant Height (cm)	Length of spike (cm)	No. of spikelet	1000-kernel Weight (g)	Mean IT/DS for Bgt	Mean IT/DS for Pst
MY11	82.5 ± 1.6b	8.5 ± 0.5c	17.6 ± 0.6b	36.4 ± 1.1b	4/95	4/90
HH41	99.0 ± 5.8a	10.0 ± 0.4a	20 .8 ± 1.9a	37.5 ± 0.9b	0;/5	0/0
M26-B	94.0 ± 5.4a	11.0 ± 0.6a	20 .0 ± 2.5a	31.6 ± 1.7b	0;-1/12	0;/5
R1870-C	93.0 ± 4.3a	10.5 ± 0.5a	19.3 ± 2.1a	43.2 ± 2.0a	4/85	0;/5
R522-D	68.0 ± 3.8c	6.2 ± 0.2d	17.0 ± 2.2b	16.9 ± 1.9d	1/18	4/95
R600-E	97.3 ± 6.5a	9.8 ± 0.7b	20.1 ± 1.8a	26.8 ± 0.8c	1/20	1/10
H86	93.4 ± 5.4a	9.5 ± 0.6b	20.3 ± 2.4a	27.3 ± 0.7c	1/25	4/80
H136	84.5 ± 2.7b	8.4 ± 0.8c	16.9 ± 1.2b	33.7 ± 1.2b	4/90	1/10

*Values with the same letter in the same column do not differ significantly at *P* < 0.05

IT and DS means the Infection Type and disease severity, respectively

The 6R^afr^ lines were resistant when challenged with a *Pst* race mixture (CYR32, CYR33, and CYR34) in the field. In the field tests, HH41, M26-B, r1870-C, and r600-E were resistant, which had scores of IT 0–1, mean DS = 4.08 (0–10), and the average rAUDPC with mean 27.6%,(22.1 to 33.1%). The wheat parent MY11 and lines r522-D and H86 were susceptible with scores of IT = 4 and mean DS = 89.5 (80–95) (Table 1, Fig. 6b). It suggested that *S. africanum* 6R^afr^S carries a gene(s) for stripe rust resistance in the S3 to S4 segment (~FL 0.90–1.00).

### Evaluation of agronomic traits

Agronomic traits of the wheat- *S. africanum* lines were evaluated under field conditions during the 2015 to 2018 growing seasons. As shown in Fig. 5c, lines HH41, M26-B, r600-E, r1870-C had increased spike length, plant height, and spikelet number relative to parent MY11 (Table 1). The data indicated that 6R^afr^ had little linkage drag on agronomic traits in the wheat background. However, line r522-D which lacked of the terminal pSc119.2-rich region of 6R^afr^S was significantly inferior in spike length and spikelet number. These results suggested that beneficial genes related to spike development might be located in the deleted segment of chromosome 6R^afr^S in r522-D. Line r1870-C displayed higher 1000-kernel weight than MY11 and the other 6R^afr^ lines, indicating that regions L4 to L5 in 6R^afr^L might have a negative effect on grain weight.

## Discussion

Molecular markers covering the entire rye genome and distributing evenly across different chromosome regions is helpful for detection of introgressed alien chromatin in wheat [21]. Qiu et al. [33] developed 578 rye-specific PCR markers and Li et al. [15] produced 300 6R^Ku^L-specific markers by using high-throughput SLAF-seq technology. The aforementioned markers were used for identification of chromosomes 1R^Ku^ to 7R^Ku^ and their specific arms in wheat. Bauer et al. [36] obtained a de novo assembled 1.68 Gb of gap-free sequence representing 21.26% of the 7.9 Gb genome of inbred winter rye line Lo7. The assembled pseudo-molecules of the Lo7 genome provide relative physical locations of rye-specific markers. Du et al. [15] developed 16 specific markers for a 6R^Ku^L mini-chromosome and found that 14 of the 16 sequences had 91–100% similarity with nine scaffolds derived from chromosome 6R^Lo7^ [36]. In the present study, 66 SLAF markers specific for rye 6R^Ku^ were mapped on *S. africanum* 6R^afr^. Of these markers 16 (24%) were physically located on the 6R^Lo7^ pseudo-molecule maps. The PCR markers (Fig. 4) and RNAseq data (Fig. 5) blasted against the Lo7 genome sequences provided evidence for localization of deletion breakage points on the 6R^afr^L in line r1870-C. A complete, high-resolution genomic sequence of rye would be an invaluable resource for generating more chromosome-region-specific markers for targeting introgressed chromatin from different cultivated and wild rye genomes.

Well-conserved genome collinearity among cultivated and wild rye species is evident from the presence of large syntenic blocks that were disrupted by chromosomal rearrangements [31, 39, 40]. In the present study, a length differences between 6R and 6R^afr^ was evident in a hybrid between HH41 and DS6R. This length difference might be due to large accumulations of rye-specific repeats like the pSc200 sequence, in both the terminal region of 6RS and sub-terminal region of 6RL. Chromosome 6R^afr^ lacked all Oligo-pSc200 signals. A molecular marker analysis confirmed the complex segmental rearrangement from chromosome 6RS to 2RS in cultivated rye [40, 41], whereas *S. africanum* 6R^afr^S appears to have maintained close collinearity to the short arms of homoeologous group 6 chromosomes from the common ancestor of rye and wheat. Moreover, the peri-centromeric regions between 6R^Ku^L and 6R^afr^L appeared well conserved, but the sub-telomeric regions showed higher structural variation. The greatest divergence of 6R^Ku^L and 6R^afr^L was in the telomeric regions (Fig. 5). Data from comparative chromosome-specific PLUG and CINAU marker analysis suggested that the syntenic regions among wheat homoeologous group 6, and proximal sections of 6R and 6R^afr^ of rye were conserved, but the sub-terminal and telomeric regions of 6RL and 6R^afr^L were largely divergent.

Rye chromosomes with loss of telomeric C-bands or deletion of large segments have been described [42, 43]. Spontaneous redistribution and deletion of telomeric heterochromatin on rye chromosome 6R, and the complex structure of the distal region of 6RL, might have reduced the level of recovery of wheat-rye recombinants [44]. Recently, Hao et al. [16] found that cultivated rye cv. QL chromosome 6R gave rise to different spontaneous deletions and translocations when it was transferred into wheat. Du et al. [15] identified a 6R^Ku^L mini-chromosome addition line, MiA6R^Ku^L, among the self-pollinated progeny of a wheat-rye 6R^Ku^L monotelosomic addition line. In the present study, several types of 6R^afr^ telosomes were also observed among the offspring of the hybrid between HH41 and MY11 (Fig. 3). A strong pSc119.2 accumulated telomeric regions of 6R^afr^S was deleted in r522-D, whereas an inversion involving the distal region of 6R^afr^L appeared to have occurred in r600-E (Fig. 2).

Chromosome 6R from different cultivated rye genotypes contains disease resistance genes with potential for wheat improvement. 6RL from rye cv. Prolific [9, 10], JZHM [13], German White [11] and Kustro [14] have powdery mildew resistance genes. Hao et al. [16] reported powdery mildew resistance gene *Pm56* on 6RS from rye cv. QL, and Li et al. [18] located stripe rust resistance gene *Yr83* on 6RL. In our present study the L4 to L5 segment of chromosome 6R^afr^L conditioned adult plant resistance to powdery mildew, and 6R^afr^S carried a gene(s) for resistance to stripe rust. It is thus noteworthy that further introgression of chromatin from alternative cultivated and wild rye germplasm may provide additional disease resistance genes for wheat improvement.

## Conclusions

In the present study, we developed and identified new wheat-*S. africanum* chromosome 6R^afr^ deletion and translocaton lines by using sequential in situ hybridization and molecular markers analysis. The large chromosome structural rearrangement between *S. africanum* 6R^afr^ and *S. cereale* 6R may be occurred during the evolution and speciation of Secale genus. The wheat- *S. africanum* 6R^afr^ lines displayed novel resistance to a powdery mildew and stripe rust at adult plant stages. We found that the gene(s) on the long arm of 6R^afr^ at FL0.85–1.00 were responsible for powdery mildew resistance, while the gene(s) was located in the terminal region of 6R^afr^S at FL0.95–1.00 were comply for stripe rust resistance. The wheat-*S. africanum* 6R^afr^ introgression lines with superior agronomic traits may serve as new bridging germplasm for wheat breeding.

## Methods

### Plant materials

The wheat-*S. cereale* 6R(6D) substitution line (DS6R) was provided by Dr. Ian Dundas, University of Adelaide, Australia. The *S. cereale* cv. QL, wheat cv. Mianyang 11 (MY11), wheat - *S. africanum* 6R^afr^(6D) substitution line HH41 [29], and the *T. durum*-*S. africanum* amphiploid (YF) are maintained in the School of Life Science and Technology, University of Electronic Science and Technology of China. Wheat-*S. africanum* 6R^afr^ deletions were selected from M_4_ generation lines induced by ^60^Co γ-rays irradiation at the Institute of Biological and Nuclear Technology, Sichuan Academy of Agricultural Sciences in Chengdu. The wheat-*S. africanum* 6R^afr^ translocation and telosomic lines were obtained from the F_4_ populations derived from a cross of HH41 and MY11.

### Fluorescence in situ hybridization (FISH)

Seedling roots for FISH were collected when they were 2-3 cm long and treated with nitrous oxide gas for 2 h under 1.0 MPa pressure. The treated roots were fixed in 90% acetic acid and washed before they were digested in the solution of 2% cellulase and 1% pectolyase (Yakult Pharmaceutical, Tokyo), which was referred the study of Lang et al. [38]. The digested root sections were washed and the meristematic portions were mashed to form a cellular suspension in 100% acetic acid. The cell suspensions were dropped onto glass slides for chromosome preparation according to Han et al. [45]. The oligo-nucleotide probes Oligo-pSc200, Oligo-pSc119.2 and Oligo-pTa535 were used for identifying the wheat chromosomes following the descriptions of Fu et al. [30]. The production of the labeled oligonucleotide probes and the protocol of non-denaturing FISH (ND-FISH) employing synthesized probes was described by Tang et al. [46]. After ND-FISH, the chromosome squashes for sequential FISH were washed twice to remove hybridization signals [18]. The photography of FISH signals was performed under an fluorescence microscope (BX53, Olympus), and the images were processed according to Lang et al. [38].

### Molecular marker analysis

DNA was extracted from young leaves using a sodium dodecyl sulfate (SDS) protocol [29]. Primers from specific length amplified fragment sequencing (SLAF-seq) based PCR markers in rye [33] and 6R-specific markers [14, 15] were provided by Dr. Shulan Fu, Sichuan Agricultural University, Chengdu. PCR-based Landmark Unique Gene (PLUG) primers [34, 41], CINAU (Cytogenetics Institute, Nanjing Agricultural University, Nanjing, China) primers [35], and rye EST-derived SSR primers [31] were synthesized by Shanghai Invitrogen Biotechnology Co. Ltd. Polymerase chain reaction amplification, restriction enzyme-digestion and electrophoresis were as described by Li et al. [28]. Markers for physical location in chromosomes were obtained by searching the database from the International Wheat Genome Sequencing Consortium whole genome assembly ref. v1.0 [37], and the whole-genome shotgun sequencing assembly of inbred winter rye line Lo7 [36].

### RNA-seq and transcriptomic analysis

Leaves of two-week-old seedlings were harvested for RNA extraction and sequencing. Total RNA was isolated and quality tested as previously described [47]. The cDNA library construction and sequencing were performed with a HiSeq2000 analyser (Illumina) according to the manufacturer’s instructions. Transcriptome assembly was accomplished using Trinity [48] with default parameters. All unigene sequences from RNA-seq that had the highest identity to the wheat and rye chromosome homologous group 6 were selected for differential expression analysis. The reference genome sequence of Chinese spring (CS) was downloaded from IWGSC (http://www.wheatgenome.org/) [37]. The draft genome sequences of rye was from GrainGenes (https://wheat.pw.usda.gov/cgi-bin/seqserve/blast_rye.cgi) [36]. Four RNA-seq datasets from wheat-6R^afr^ lines and MY11 wheat were compared for genome and gene expression analyses.

Powdery mildew and stripe rust reactions.

Lines were evaluated at the seedling to adult plant stages in a greenhouse at 22 °C and photoperiod of 14 h of light per day. A local mixed *Bgt* isolates was used for inoculation. Four-leaf stage was found to be suitable for measuring APR, and the APR response of each plant was recorded on infection type (IT) and disease severity (DS) scale at 10, 15 and 20 days post-inoculation Molher et al. [49]. The two superposed leaves below flag leaf showing the highest infestation in the parent MY11 were examined as control.

Stripe rust reactions were observed in field-grown plants at the Sichuan Academy of Agricultural Science Experimental Station. Ten plants were grown 1 m in rows with a 25 cm spacing between rows. Bread wheat MY11 planted on both sides of each experimental row served as an inoculum spreader and susceptible control after inoculation with races CYR32, 33 and 34. Reactions evaluated at the heading and grain-filling stages were scored for adult plant responses based on the infection types (IT) and disease severity (DS), the area under the disease progress curve (AUDPC) and relative AUDPC (rAUDPC) according to the report of Yuan et al. [50].

Evaluation of agronomic traits.

Each line was evaluated for plant height (cm), spike length (cm), spikelets per spike, and 1000-kernel weight (g). The significance of differences in agronomic traits among the lines was assessed using SPSS 16.0 (IBM, Armonk, NY, USA).

## Supplementary information

**Additional file 1: Supplementary Table 1.** Primers generated 6R^afr^ specific amplification and their physical location of Lo7

## Data Availability

The datasets of raw Illumina sequences generated in the current study were deposited to the National Center for Biotechnology Information (NCBI) and can be accessed in the Short Read Archive (SRA) database (https://www.ncbi.nlm.nih.gov/sra) as accession number PRJNA610716. All plant materials analysed during this study are available from the corresponding author by request.

## Abbreviations

APR: Adult-plant resistance
AUDPC: Area under the disease progress curve
Bgt: *Blumeria graminis* f. sp. *tritici*
DAPI: 4–6-diamino-2- phenylindole
DS: Disease severity
FAM: 6-carboxyfluorescein
FISH: Fluorescence in situ hybridization
IT: Infection type
ND-FISH: non-denaturing fluorescence in situ hybridization
Pst: *Puccinia striiformis* f. sp. t*ritici*
SLAF: Specific length amplified fragment
SDS: Sodium dodecyl sulfate
Tamra: 6-carboxytetramethylrhodamine
