# Supplementary material for: Molecular dissection of Secale africanum chromosome 6Rafr in wheat enabled localization of genes for resistance to powdery mildew and stripe rust
Source: BMC Plant Biol. 2020 Mar 31;20:134. doi: 10.1186/s12870-020-02351-1 (PMC7106737; doi:10.1186/s12870-020-02351-1)
Supplement: Supplementary file 1 — Additional file 1: Supplementary Table 1. Primers generated 6Rafr specific amplification and their physical location of Lo7 [file 12870_2020_2351_MOESM1_ESM.docx]

**Supplementary Table 1**  Primers generated 6R^afr^ specific amplification and their physical location of Lo7

| Marker | Forward (5'-3') | Reverse (5'-3') | 6Ra product | Lo7 Location (Mb)* |
| --- | --- | --- | --- | --- |
| GRM964 | GGGGCTAAACAGTAGCAGATG | TCCATCATGCATCAGCTGTAA | 220bp | - (2R) |
| 6VS-Bd1 | CAGGTGTTGGTTCAGTGCTCA | CCTGCCATAGTGTCGAAATCTATG | 350bp | - (2R) |
| KU791 | ACCTGCATGCATACCAATGA | GACGGTGCATGTTTGATGTC | 400bp | - |
| KU.903 | CGATAACAGCATCCCAAGGT | AAAGCCCAAACACACAGCTC | 400bp | 7.6 |
| 6VS-Bd2 | TCATGCTTTAGCTGAGTTTGACCA | GAACTTCCACAGCTTCTCATTTGA | 400bp | 9.7 |
| CINAU1557 | TCGACATTCAGTGATGGTGATC | TTGCCATCCCTGTACAGAGG | 300bp | 17.9 |
| CINAU1509 | CTCTTGGGTCATACTGCTCCT | ATGCACGACAATACAATGAAGAA | 650bp | 21.5 |
| CINAU1505 | AGACAACTTTGGTGGATTCAATG | TCCTTTCCTGCACAACTTGA | 1050bp | 24.4 |
| KU.701 | GCATGGATAAACCAGAGACAAA | TGGCATTAGATCTGATTTGAGGT | 400bp | 26.8 |
| KU.423 | GCTCCTCTGAAAATGCTGCT | CATGGCAAGTTCACAGTATTGG | 400bp | 30.4 |
| KU-880 | GCACCTCCCCAGGATCTAGT | TCTTGCGGTGTTGACAGAAG | 400bp | 25.2 |
| KU-825 | TCAGTGCAGCCTTGACATTT | TTTGACAGGCTTTGAGGTGA | 400bp | 33.8 |
| KU555 | GGTGCAGGGATGTAGAGAGG | AGGAAGCGCTACATGGGTCT | 400bp | - |
| KU226 | GTGACGATTTCGACGATCAG | CTCAGTGGTGCCAACTGTGT | 400bp | - |
| KU-259 | TGTGTGTGCGTTGGTCTACA | CGTCGTTGGATGGTTTAGAA | 400bp | 42.8 |
| CINAU1503 | GCAGCCCACTAAAGCCTTAC | AATACAACCCAACGACCACA | 950bp | 43.2 |
| KU-55 | CAGACGAAGCATAATTACCA | TTCGTCCCTTTGATTACTTG | 400bp | 45.4 |
| KU-175 | GGCAATTGTCCATGGTTAAAA | CTACCTCCCTTCGCTAATCG | 300bp | 56.9 |
| KU-87 | TTTTGAGTTGATTGCCCTGA | CGCCGACAATGTTTTCCTAT | 400bp | 57.6 |
| CINAU1558 | GGACAGCGATGTATGCCAAA | GCAGAACTGGTTTGCTGTCA | 650bp | 71.1 |
| TNAC1685 | ATGGATAGCGGAAGCGACTC | AGCGTTTCCTCCGGTCTT | 800bp | 73.9 |
| KU-976 | GTCCAGCTACAACGGCTCTC | TAGCCTCGAGAAGGGTCAAA | 400bp | 75.8 |
| KU-801 | CAATAGCCCAGTCGACAACC | ATAAGCCAGCTCGTGGAGAA | 400bp | 84.4 |
| CINAU1515 | ATGCACATGGACGTCGTTC | GGCCCCTAAGCTTTTCCTTG | 450bp | 85.2 |
| CINAU1504 | GCTGATCTGGATGTTCTTGACC | GCTGCTGAGGGGATGGTAAT | 450bp | 86.1 |
| KU-841 | GCTATGGGCTGAGTGTCTCC | TCAAGGCTTCCTCCTCAACT | 500bp | 86.1 |
| TNAC1740 | CGGAAGTGCTCGATTGTATCT | GCGGGTTTCTTCTCAACCTT | 680bp | 93.0 |
| CINAU1561 | TTGGCTATTATGTAGGGGTTGAT | TTGATCCGAGTCGCCGTTAT | 950bp | 95.7 |
| CINAU1522 | CAGCAGCAGGTTGAGGCA | GGTGATTGAGGACGCAGAAG | 480bp | 97.0 |
| KU-311 | TGGTAGTAAAGTAACTGTGCAA | GAATCATTGAATTATCACGAGC | 400bp | 98.7 |
| CINAU1563 | CCTACTGCACTTGTTCCACA | GCAATGTCTGGATAACAACTGC | 300bp | 99.7 |
| TNAC1748 | TCGTAGAATTGGTCGACGATG | ATGGATTGGCAAAGAAAGATG | 750bp | 100.9 |
| CINAU1517 | GAAGCTCTGGAATCATGGCG | CATGCCAGTTGAACTCCAGG | 450bp | 102.4 |
| CINAU1510 | TGCCGTGATAATTGATGCCA | TTCTGTTGTGGAAGTTGCGG | 700bp | 105.8 |
| KU-430 | CAAAAGAGGGAAGCTTGGAG | TTCCTTTCAGTGGATGACCTG | 400bp | 107.9 |
| CINAU1530 | TTTGAGAAAGAACCATGGAAAGG | AAGATCCCTGGCACGATTCT | 300bp | 116.9 |
| KU-384 | TGTTTCCCCTCCCGTTATAG | GGGAAGGTGATTGATGTTGG | 400bp | 118.5 |
| CINAU1523 | TGCAGAGGATTTTAAATGGAAGG | ACACTTCCACTCTTTCCAGGA | 650bp | 125.8 |
| CINAU1495 | TGATCTAGCTTTTACTCCAGACG | AGACTGCACCATCAAGTCCA | 550bp | 125.9 |
| KU-322 | CTGGATATGTGCGTTTCGTG | CTCGGCGACCTTACACTTTC | 400bp | 137.1 |
| KU-416 | CTAACGCTTTCGAGCTATCG | CATGCACGACTTTTAGGGTTT | 400bp | 147.8 |
| KU-10 | ATCGACCAACTCACGAGCA | GGGAGGAGAGGAAAGCAGAT | 400bp | 149.2 |
| KU-340 | CGTGCCGTCCTAGTTTTCAT | GCACATCGAGCTTGATACACA | 400bp | 152.1 |
| CINAU1600 | AGAGTGTGATCCTCTTGTGCA | ACCAGCATAGGAAGATAGGGT | 550bp | 154.2 |
| TNAC1953 | CTCATAAGACCACCAACCTTCA | GTGCCTTTCGGGAAGAACTC | 700bp | 158.3 |
| TNAC1848 | AAATACACCCGATCAGCATCA | GAGGAGTGTGAGAGCCTCCTT | 260bp | 158.7 |
| TNAC1844 | CTTTATGCGCGGTAACACATT | TTGAAGTTTGGGATCAACACA | 300bp | 164.9 |
| CINAU1591 | TGTGTACCGGGAATGGGC | TTCCAGTTGTTGAAGTCACCA | 650bp | 165.0 |
| CINAU1575 | GGCTGTGACTGGCATGAC | GATCAACTCCACCGTTTGCC | 800bp | 168.6 |

Note: The SLAF-seq based markers KU and KU- are from Li et al. (2015) and Qiu et al. (2016), respectively. The CINAU markers are from Zhang et al. (2017), the TNAC markers are courtesy of Ishikawa et al. (2009); the GMR964 markers are from Martis et al. (2013). The 6VS-Bd primers are from He et al. (2013). * indicates that the first three markers were physically located on 2R^Lo7^, while the remaining markers are located on 6R^Lo7^.
